# Damage Mechanism of HgCdTe Focal Plane Array Detector Irradiated Using Mid-Infrared Pulse Laser

**DOI:** 10.3390/s23239370

**Published:** 2023-11-23

**Authors:** Yin Zhang, Changbin Zheng, Yang Liu, Yunzhe Wang, Yongbo Xu, Junfeng Shao

**Affiliations:** 1 State Key Laboratory of Laser Interaction with Matter, Changchun Institute of Optics, Fine Mechanics and Physics, Chinese Academy of Sciences, Changchun 130022, Chinaliuyangdk@ciomp.ac.cn (Y.L.);; 2 University of Chinese Academy of Sciences, Beijing 100049, China

**Keywords:** mid-infrared laser, HgCdTe FPAs detector, irradiation effect, damage threshold

## Abstract

To investigate the damage threshold and mechanism of a mid-infrared HgCdTe focal plane array (FPA) detector, relevant experimental and theoretical studies were conducted. The line damage threshold of a HgCdTe FPA detector may be within the range of 0.59 Jcm^−2^ to 0.71 Jcm^−2^. The full frame damage threshold of the detector may be in the range of 0.86 Jcm^−2^ to 1.17 Jcm^−2^. Experimental results showed that when the energy density reaches 1.17 Jcm^−2^, the detector exhibits irreversible full frame damage and is completely unable to image. Based on the finite element method, a three-dimensional model of HgCdTe FPAs detector was established to study the heat transfer mechanism, internal stress, and damage sequence. When HgCdTe melts, we think that the detector is damaged. Under these conditions, the theoretical damage threshold calculated using the detector model is 0.55 Jcm^−2^. The difference between theoretical and experimental values was analyzed. The relationship between damage threshold and pulse width was also studied. It was found that when the pulse width is less than 1000 ns, the damage threshold characterized by peak power density is inversely proportional to pulse width. This relationship can help us predict the experimental damage threshold of an FPA detector. This model is reasonable and convenient for studying the damage of FPA detectors with a mid-infrared pulse laser. The research content in this article has important reference significance for the damage and protection of HgCdTe FPA detectors.

## 1. Introduction

HgCdTe is a direct bandgap semiconductor material that is formed via the combination of CdTe and HgTe, resulting in a ternary compound [1]. The bandgap of HgCdTe can be adjusted within the range of 0.1–1.5 eV by altering the composition ratio of Cd, which enables its band to cover the crucial atmospheric infrared window [2]. As a result, HgCdTe detectors have found extensive applications in space exploration, target tracking, and environmental monitoring. At present, HgCdTe detectors have developed to the third generation. In comparison to the first generation, which primarily comprised PV and PC types, focal plane array (FPA) detectors have become a typical representative of second-generation HgCdTe detectors owing to advancements in surface passivation and semiconductor processing technology. The third-generation HgCdTe detectors [3] were built upon the second generation and are progressing towards small pixels [4], high-temperature operation [5,6], and multi-color detection [7,8]. HgCdTe FPAs detector exhibits a robust ability to detect weak light signals. For this reason, it is confronted with the risk of being extremely vulnerable to laser interference and damage.

Research on laser-induced damage to Hg_1−*x*_Cd_x_Te crystals and detectors began as early as the 1970s. The research results of Bartoli’s team [9] are the most representative. They not only carried out corresponding experimental research but also established a one-dimensional, semi-infinite, solid model uniformly irradiated with a laser. The model has obvious advantages and rationality in calculating the damage threshold of PV- or PC-type detectors. They took into account the micro-layer structure of PC-type HgCdTe detectors and the Gaussian optical spot, finally obtaining a new model with a wider range of applications [10]. Arora et al. studied the responsivity of a PC-type HgCdTe detector irradiated with CW CO_2_ [11]. They found that the responsivity decreased by two orders of magnitude when the temperature of the detector increased from 77 K to 150 K. Zhao et al. researched the interaction between a CW CO_2_ laser and P-type HgCdTe crystals [12]. They established a two-dimensional damage model solved using the finite difference method. Cai et al. carried out a fracture damage experiment of Hg_0.8_Cd_0.2_Te crystal and found that thermal stress was the main reason for crystal fracture [13]. Tang et al. carried out experimental and theoretical research on Hg_0.714_Cd_0.286_Te crystal damaged using a repetitive pulsed laser [14]. They found that the damage threshold of Hg_0.714_Cd_0.286_Te crystal was independent of the repetition frequency. Chen et al. used a Nd: YAG pulsed laser to damage HgCdTe crystal at room temperature [15]. The wavy periodic structure was scientifically explained using the theory of transverse surface acoustic wave. Jia’s research team introduced a method of using combined pulse lasers to damage or process materials [16,17]. They gave us an important inspiration: using a combined pulse damage detector may effectively reduce the damage threshold of the detector.

A summary of past research can be found in the following points.

1. Research subjects include mostly Hg_1−x_Cd_x_Te crystal and PV-type or PC-type HgCdTe detectors with working wavelengths located in LWIR. The laser wavelengths used in damage experiments are mostly 10.6 μm or 9.3 μm.
2. Research directions mainly include detector response rate, damage threshold, and damage morphology analysis.
3. In theoretical research, one-dimensional or two-dimensional models are often constructed based on the structure of PV- or PC-type detectors.

In summary, there have been few experimental and theoretical studies on the damage of HgCdTe FPA detectors irradiated with mid-infrared lasers. Considering the significant differences in the structure between FPA and PV/PC detectors, the model of the first-generation detector is no longer fully applicable to FPA detectors. Therefore, it is necessary to establish a new damage model for HgCdTe FPA detectors. In terms of research content, attention is not only paid to the damage threshold but also to the internal heat transfer mechanism, thermal stress and deformation, damage sequence, pulse width factors, and other issues facing FPA detectors. There are also significant differences in the application fields of LWIR and MWIR. LWIR can be used for non-destructive testing and early cancer diagnosis. In addition, LWIR can also be used for optical technology research on the precipitation of biological tissues such as proteins and red blood cells. MWIR is widely used in industrial detection, military reconnaissance, environmental monitoring, and other fields. In the field of mid-infrared lasers, DF laser is one of the few lasers that can output high energy. It has significant value in the military field. The most common output wavelength of a DF laser is 3.9 μm. Therefore, this article focuses on the damage effect of a 3.9 μm pulse laser on HgCdTe FPA detectors.

We first conducted experimental research on the damage caused to HgCdTe FPA detectors by mid-infrared pulse laser. The experimental damage threshold *E*_0_ of the detector was obtained. A three-dimensional finite element model of the FPA detector was established. The temperature and stress characteristics of the detector during laser irradiation were studied from the perspectives of heat transfer and solid mechanics. This model can also provide the theoretical damage threshold *E_c_* of a detector under different pulse widths, which is basically consistent with *E*_0_ under the same conditions, verifying the reliability of the model. These topics have important reference significance for the structural optimization, damage mechanism, and protection of detectors.

## 2. Materials and Methods

### 2.1. Experimental Setup

**Figure 1:**
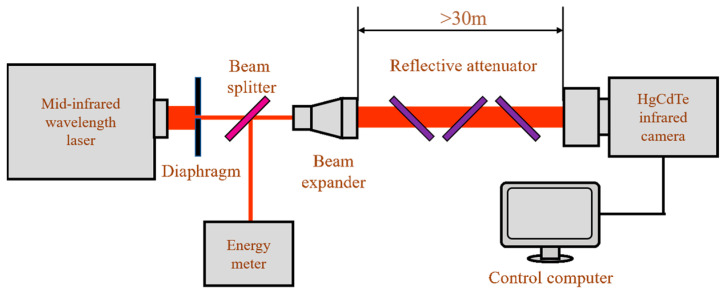
Schematic diagram of experimental setup.

Experimental research on a HgCdTe FPAs detector irradiated with a mid-infrared pulse laser was conducted, and the detector damage threshold was obtained. The experimental setup is shown in Figure 1. The experimental light source was a DF laser, with an output pulse width of 30 ns. Its central wavelength was 3.9 μm, which was exactly within the response band of the HgCdTe FPAs detector. After passing through a 10× beam expander, laser divergence angle was compressed to 0.4 mrad. The distance between the beam expander and the camera lens exceeded 30 m, ensuring that the spot size before reaching the camera was greater than the diameter of entrance pupil. The HgCdTe infrared camera used in the experiment was a commercially available camera developed by the 11th Research Institute of China Electronics Technology Group Corporation (Beijing, China), which included an infrared lens and a HgCdTe FPAs detector. Stirling compression refrigeration provided a low temperature working environment for the detector. The pixel scale of the FPA detector was 320 × 256. The distance between adjacent pixels was 30 μm.

### 2.2. Theoretical Model

#### 2.2.1. Model Structure

**Figure 2:**
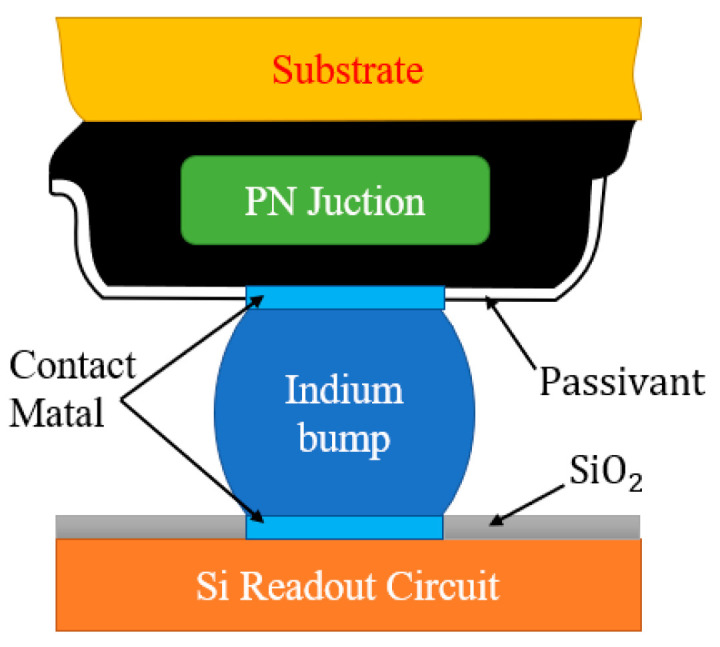
Schematic diagram of HgCdTe FPA detector chip structure.

The typical structure of a HgCdTe FPA detector is shown in Figure 2. The detector chip comprises a Si readout circuit situated at the bottom layer, which serves to process the current generated in the photosensitive unit. An indium column is connected to both ends of the Si readout circuit and PN junction through connect metal, with the height of the connect metal being significantly smaller in comparison with that of the indium column. In the absence of an indium column, the surface of the Si readout circuit is covered with very thin SiO_2_ and epoxy, which play roles in insulation and fixation. Each PN junction of the HgCdTe layer is indirectly connected with the indium column. There are barrier layers and passivation layers around the PN junction to divide pixel units and reduce leakage of the current. The top of the chip has growth substrate composed of HgCdTe. CdZnTe is the ideal material for growing HgCdTe, as its lattice can be perfectly matched by adjusting the composition ratio of Cd. Si and GaAs can also be used as growth substrates, which typically require several buffer layers [18].

In order to facilitate calculation, the structure of the detector was reasonably simplified on the premise of ensuring rationality of the model. This was accomplished through the following assumptions:

1. Layers that are relatively thin, such as the connect metal layer, the passivation layer, and the insulation layer, are ignored. The thickness of these layers is less than one-tenth of that of the photosensitive layer. Ignoring them does not have a negative impact on the calculation results but instead helps reduce the difficulty of mesh generation and improves the mesh quality.
2. The thicknesses of the Si readout circuit layer and the CdZnTe layer are reduced. The Si layer is directly connected to the refrigeration module, and its temperature can always be considered 77 K. It is also reasonable to decrease the thickness of the CdZnTe layer due to its high transmissivity to the response wave band. The absorption and reflection occurring in the CdZnTe layer are ignored.
3. Inter-layer absorption and reflection re disregarded. It is though that the laser directly reaches the HgCdTe layer through the substrate layer.
4. Unlike previous models that chose material physical parameters as a constant, the core parameters of this model are set as functions of temperature, such as absorption coefficient, thermal conductivity, and heat capacity.
5. All layers are regarded as isotropic crystals.

**Figure 3:**
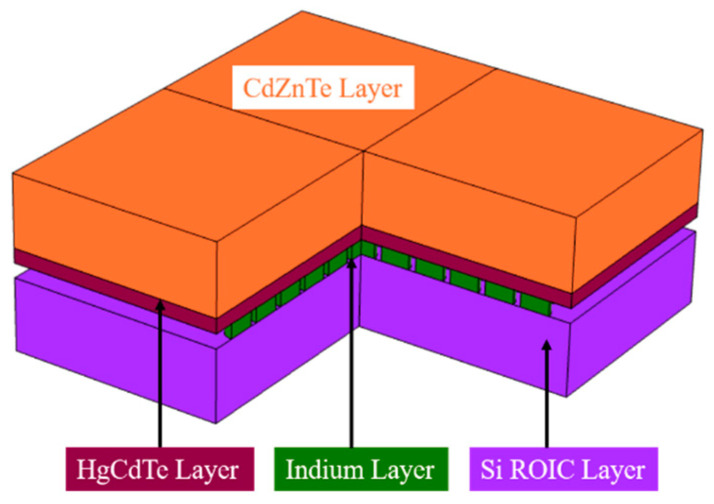
Three-dimensional model of HgCdTe FPA detector.

In Figure 3, the HgCdTe FPA detector structure is simplified into a four-layer model, which is composed of Si, an In column, HgCdTe, and CdZnTe from bottom to top. They represent the readout circuit layer, connection layer, photosensitive layer, and substrate layer, respectively. The thickness of the CdZnTe layer is 50 μm, with dimensions of 300 μm in both length and width. HgCdTe layer has a height of 10 μm and has dimensions identical to the CdZnTe layer. The indium column has a height and radius of 10 μm, with an interval of 30 μm between adjacent columns. The Si layer is identical in size to the CdZnTe layer. In order to reduce the computational time, only a quarter of the model was retained using symmetric thermal boundary conditions.

#### 2.2.2. Physical Equation

In order to conduct thermal analysis of the model, it is imperative to solve the solid heat transfer equation to obtain the temperature distribution function *T*(*x,y,z*).

(1) $$\rho c(T)\frac{\partial T}{\partial t}=k(T)\left(\frac{\partial^{2}T}{\partial^{2}x}+\frac{\partial^{2}T}{\partial^{2}y}+\frac{\partial^{2}T}{\partial^{2}z}\right)+Q$$

where ρ, c(T), and k(T) are density, heat capacity, and thermal conductivity, respectively. Q is the heat absorbed per unit volume per unit time, which is directly related to the absorption coefficient of the material. Considering that the model structure is very thin, and the absorption coefficient of HgCdTe crystal is significantly high, Q can be expressed as a volume heat source. The expression is as follows:

(2) $$Q=I_{0}(1-R)\alpha (T)exp(-\alpha (T)z)$$

where $I_{0}$, α(T), and R are power density, absorption coefficient, and reflectivity, respectively. The operational temperature of HgCdTe FPAs detector is established at 77 K, necessitating the initial temperature to be set at such value. The Si layer is maintained at this temperature throughout to effectively emulate the standard function of the detector’s refrigeration unit. The initial conditions meet the following equations:

(3) T(x,y,z,0)=77K

(4) T(x,y,0,t)=77K

As a result of the minute dimensions of the chip, solely convective heat transfer between the detector surface and its ambient surroundings is taken into account. Therefore, the boundary conditions meet Equation (5), where $h_{c}$ and $T_{0}$ are the convection heat transfer coefficient and environment temperature.

(5) $$-k\frac{\partial T(x,y,z,t)}{\partial \vec{n}}=h_{c}\left(T-T_{0}\right)$$

To undertake an analysis of stress and deformation within the detector, it is necessary to solve the equilibrium differential equation.

(6) $$\rho \frac{\partial^{2}u}{\partial t^{2}}=\nabla \cdot S+F_{V}$$

(7) $$Q=-T\frac{\partial (S;\beta )}{\partial t}$$

where u, S, and $F_{V}$ represent displacement, stress, and volume force, respectively. The strain of the detector can be resolved using Hooke’s law, wherein internal prestress and viscous stress are disregarded, and only the thermal stress generated by the temperature difference in the detector is taken into account. The material is deemed as an isotropic linear elastic material, and only thermal strain $\varepsilon_{th}$ and elastic strain ε are considered. Consequently, S satisfies the following relationship:

(8) $$S=C:(\varepsilon -\varepsilon_{th})$$

(9) $$\varepsilon_{th}=\beta \left(T-T_{ref}\right)$$

where C and $T_{ref}$ are the elastic matrix and volume reference temperature. For internal displacement and deformation, it is also necessary to solve the deformation compatibility equation:

(10) $$\varepsilon =\frac{1}{2}[{\left(\nabla u\right)}^{⊺}+\nabla u]$$

#### 2.2.3. Material Physical Parameters

The material properties of each layer are shown in Table 1. Chu et al. measured the band gap Eg of Hg_1−x_Cd_x_Te crystal and gave the change rule of Eg with component *x* and temperature T [19]; see Equation (11).

(11) $$E_{g}=-0.302+1.93x+5.35\times 10^{-4}T(1-2x)-0.81x^{2}+0.832x^{3}$$

The absorption coefficient α of HgCdTe crystal includes two parts, namely, the single-photon absorption coefficient $\alpha_{l}$ and the two-photon absorption coefficient $\beta_{l}$. Krishnamurthy et al. measured the $\beta_{l}$ of HgCdTe [20]. The experimental results indicated that $\beta_{l}$ is less than 1 cm/MW for a mid-infrared laser. When $I_{0}$ is extremely high, it has a significant impact on α. The $I_{0}$ for the simulation and experiment in this paper is approximately 10^7^ Wcm^−2^. The product of $\beta_{l}$ and $I_{0}$ is less than 10^2^ cm^−1^, which is much smaller than $\alpha_{l}$. So, $\beta_{l}$ can be ignored. Chu et al. gave the expression of α of HgCdTe crystal [21]:

(12) $$\alpha =\alpha_{g}\exp[\beta_{g}(h\omega -E_{g})]$$

(13) $$\alpha_{g}=-65+1.88T+(8694-10.31T)x$$

(14) $$\beta_{g}=-1+0.083T+(21-0.13T)x$$

The specific heat of Hg_1−x_Cd_x_Te can be summarized into the following three expressions:

(15) $$c(T)=0.058T+149.76\left(50K\;<\;T<673K\right)$$

(16) $$\log c(T)=13.47{\left(\log T\right)}^{2}-76.47\log T+110.81\left(673K\;<\;T<983K\right)$$

(17) $$c(T)=292.7+1.93658\times 10^{-8}T-3.1214\times 10^{-4}T^{2}+1.53159\times 10^{-7}T^{3}\left(983K\;<\;T<1373K\right)$$

Capper gave the empirical formula of the thermal diffusion coefficient K(T) [22]. Through the relation k(T)=K(T)ρc(T), the thermal conductivity k(T) of Hg_1−x_Cd_x_Te can be obtained:

(18) $$K(T)=3.3485-12.98T+18.91T^{2}-18.933T^{3}$$

## 3. Results

In order to compare the experimental and simulation results, the pixel size of the model was consistent with our detector specifications. In addition, it was necessary to ensure the consistency of laser parameters.

### 3.1. Experimental Results

**Figure 4:**
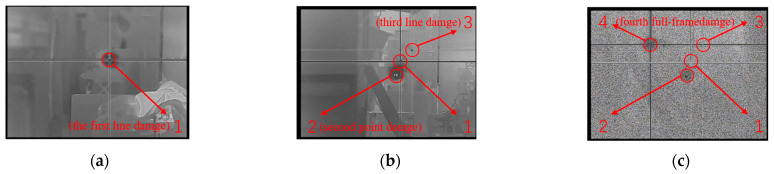
The images of the damaged camera: (**a**) the first line damage, energy density is 0.71 Jcm^−2^; (**b**) second point damage, energy density is 0.59 Jcm^−2^; third line damage, energy density is 0.86 Jcm^−2^; (**c**) fourth full-frame damage, energy density is 1.17 Jcm^−2^.

When the laser energy density reached 0.71 Jcm^−2^, line damage appeared on the detector. The damage effect is shown in Figure 4a. Keeping the order of the attenuators unchanged, the second experiment produced point damage. We determined that the output energy of the DF laser decreased due to prolonged operation. Therefore, the detector suffered point damage. The measured energy density at this time was 0.59 Jcm^−2^. Therefore, the line damage threshold may be within the range of 0.59 Jcm^−2^ to 0.71 Jcm^−2^. Increasing the energy density to 0.86 Jcm^−2^, the detector experienced line damage again. Finally, when the energy increased to 1.17 Jcm^−2^, the detector showed full frame damage and was completely unable to image normally. The full frame damage image is shown in Figure 4c. The full frame damage threshold of the detector may be in the range of 0.86 Jcm^−2^ to 1.17 Jcm^−2^. Accordingly, the experimental damage threshold *E*_0_ of the mid-infrared HgCdTe FPA detector was found to be in the range of 0.59–1.17 Jcm^−2^.

### 3.2. Simulation Results

#### 3.2.1. Heat Transfer Mechanism

**Figure 5:**
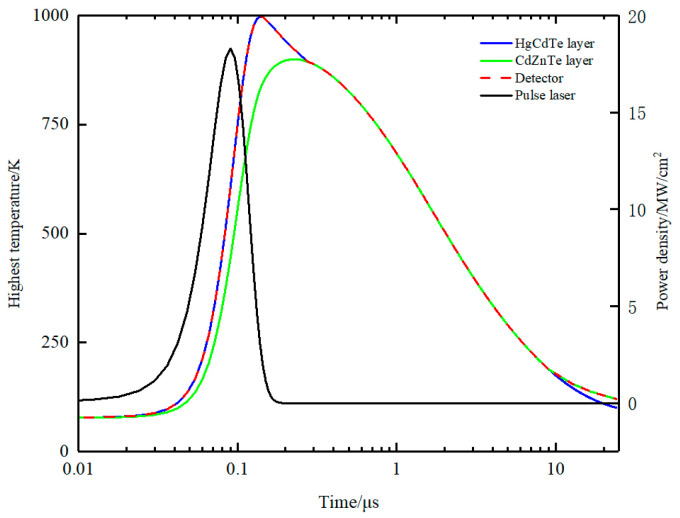
Highest temperature of detector, HgCdTe layer, and CdZnTe layer.

**Figure 6:**
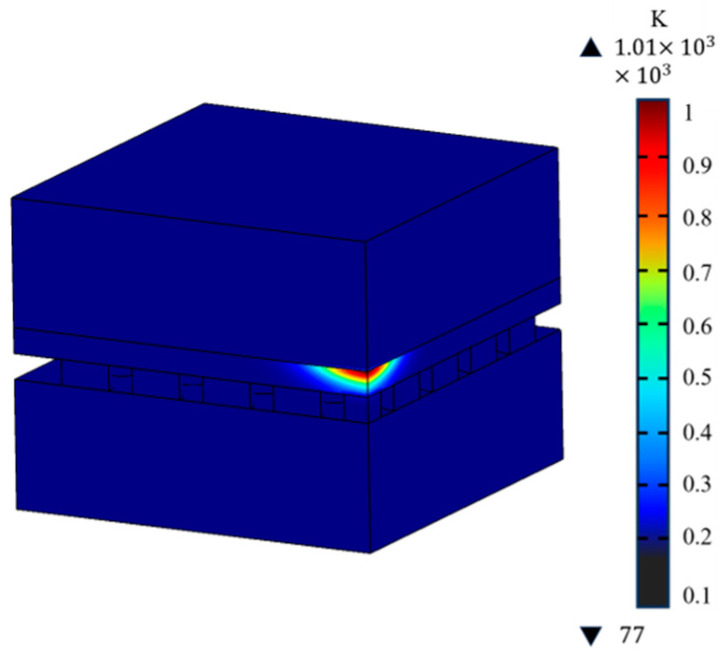
Temperature distribution of detector when the HgCdTe layer reaches the melting point.

To investigate the heat transfer process in the detector, the highest temperature of the detector, HgCdTe layer, and CdZnTe layer was monitored in real-time during the laser loading process. As depicted in Figure 5, the temperature curves correspond to the red, blue, and green lines, respectively. The black lines represent the variation in pulse laser power density with time. It can be clearly seen that temperature is mainly divided into three stages. Prior to 3 × 10^−7^ s, the red and blue lines coincide completely and are higher than the green line, indicating that the highest temperature of the detector always occurs in the HgCdTe layer. It is understandable because the HgCdTe layer absorbs laser energy, and its temperature rapidly increases. It is noteworthy that the detector temperature continues to rise until the laser power density decreases to 2.5 × 10^6^ Wcm^−2^. When the power density falls below the value, the detector temperature begins to decrease. From 3 × 10^−7^ s to 9 × 10^−6^ s, the three lines coincide completely, which indicates that the highest temperature of the detector appears on the interface of HgCdTe and CdZnTe. Therefore, as laser energy density drops, the position of highest temperature gradually moves towards the CdZnTe layer. When time exceeds 9 × 10^−6^ s, the red line and green line coincide completely and are higher than the blue line, indicating that the highest temperature appears in the CdZnTe layer. During this period, laser irradiation ceases, but the refrigerator is still working. The layer close to the refrigeration module must be cooled faster. It can be concluded that the position of the highest temperature changes over time. The HgCdTe layer absorbs the laser energy, leading to a rapid rise in temperature, while transferring heat to the surroundings. Due to the fact that the CdZnTe layer is farther from the refrigerator, the temperature rises more quickly and decreases more slowly than in the other layers. Figure 6 shows the temperature distribution of the detector when the HgCdTe layer reaches its melting point.

#### 3.2.2. Displacement Deformation and Stress

**Figure 7:**
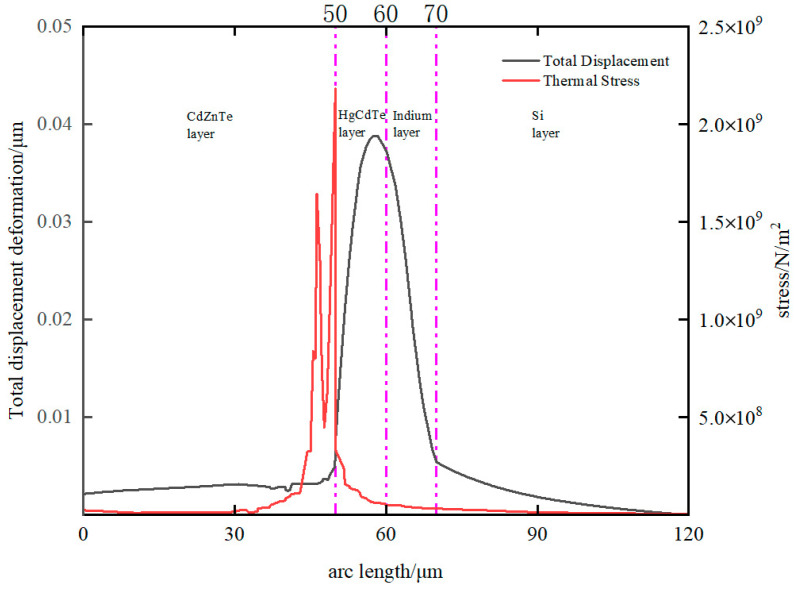
Total displacement and stress distribution on the central axis of the model.

**Figure 8:**
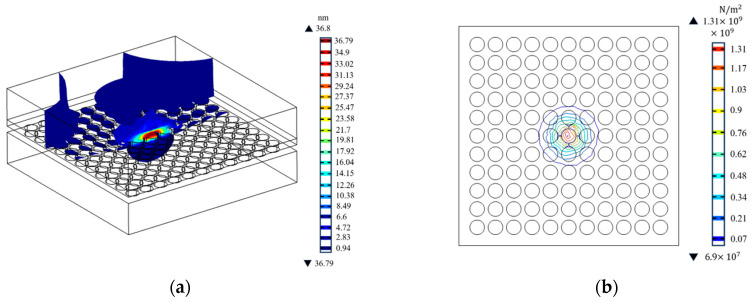
Displacement deformation and stress distribution inside the detector when HgCdTe reaches its melting point. (**a**) Isosurface distribution of displacement deformation; (**b**) stress distribution at the interface between HgCdT and CdZnTe.

An uneven distribution of temperature can generate stress and deformation internally. Through an analysis of stress and displacement deformation, it is feasible to identify the areas that are susceptible to stress damage, thereby providing valuable guidance for the construction of detectors. In Figure 7, the distribution of the total displacement deformation and stress with position can be seen. When the HgCdTe layer reaches its melting point, the maximum displacement deformation is observed in both the HgCdTe and indium layers, while the maximum stress occurs in close proximity to the interface between HgCdTe and CdZnTe. As illustrated in Figure 8a, the maximum displacement deformation inside the detector shall not exceed 40 nm, which is significantly less than the diameter of the indium column. Hence, it can be inferred that displacement deformation arising from increased temperature can be disregarded and has no discernible adverse impact on the detector’s structure. The location of maximum stress is in close proximity to the HgCdTe-CdZnTe interface, as this region experiences the highest temperature. It is worth noting that the thermal conductivity of CdZnTe is lower than that of HgCdTe. Therefore, the temperature change rate of the CdZnTe layer is significantly lower than that of the other layers. During laser irradiation, the energy transmitted from the HgCdTe layer is more concentrated near the interface of HgCdTe-CdZnTe. Slow change can prevent the thermal mismatch between CdZnTe and HgCdTe from having a significant negative impact on the interlayer structure of HgCdTe FPAs detector, which contributes to the stress near the HgCdTe–CdZnTe interface. Figure 8b shows the stress contour map of the interface. Consequently, from a stress damage standpoint, the probability of stress damage at this location is the highest.

#### 3.2.3. Damage Order and Threshold

**Figure 9:**
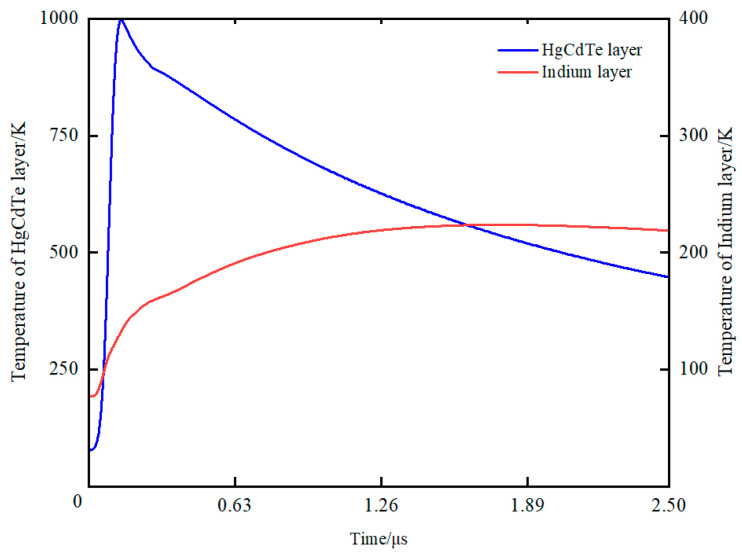
Highest temperature of HgCdTe layer and indium column layer.

Given that indium possesses a melting point that is lower than that of HgCdTe and is in intimate proximity with it, it was necessary to conduct an examination to ascertain whether the temperature of the HgCdTe layer or indium layer attains the melting point threshold first, as this is intricately linked with the mechanism underlying detector damage. The melting points of HgCdTe and indium are 993 K and 426 K, respectively. By comparing the temperature changes of the indium column layer and the HgCdTe layer, the damage order and damage threshold can be determined. According to the simulation results in Figure 9, it can be observed that the HgCdTe layer undergoes melting prior to the indium column. When the temperature of the HgCdTe layer starts to decrease, the temperature of indium continues to rise and never exceeds its melting point. If a high-energy pulse is used to irradiate the detector, indium may melt, but its melting time must be later than that of HgCdTe. Taking the melting point of the HgCdTe layer as the basis for damage determination, the theoretical damage threshold *E_c_* under the same laser parameter and pixel size was calculated using our model, which is *E_c_* = 0.55 Jcm^−2^.

Compared to the LWIR HgCdTe FPAs detector, we found that the damage threshold did not differ significantly. Zhang et al. used TEA CO_2_ to irradiate the LWIR HgCdTe FPA detector and obtained the damage threshold range of 0.69~1.23 Jcm^−2^ [23]. It can be seen that there is no significant difference in the damage threshold between MWIR and LWIR HgCdTe detectors. Regarding theoretical calculations, Bartoli’s team provided a formula for calculating the damage threshold of the PC-type HgCdTe detector. We calculated the damage threshold of the LWIR PC-type HgCdTe detector to be 0.72 Jcm^−2^. The results are still relatively close to those of the MWIR HgCdTe FPA detector model we established.

#### 3.2.4. Effect of Pulse Width on Threshold

**Figure 10:**
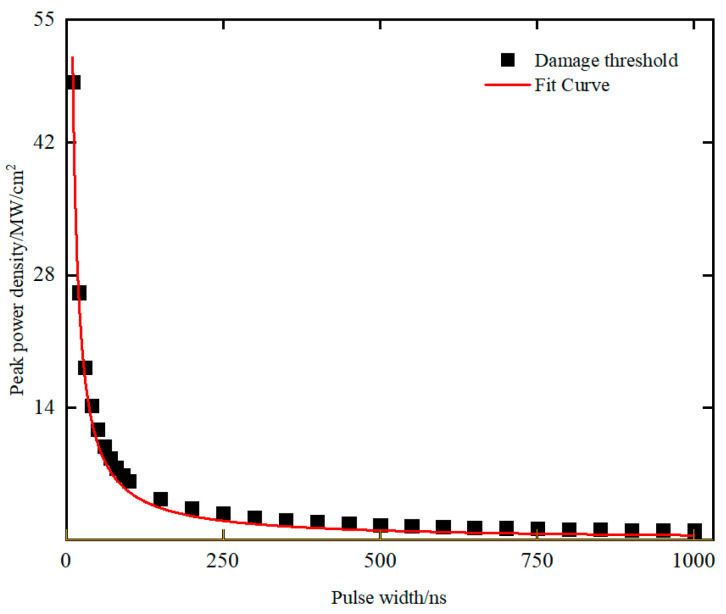
Curve of power density versus pulse width.

Bartoli’s team proposed in 1975 that the damage threshold of PV-type HgCdTe detectors is inversely proportional to the pulse width when pulse width is less than 1 μs [10]. The corresponding energy density is close to constant. It is worth exploring whether the conclusion is applicable to FPA detectors. The damage threshold within a pulse width range of 10 ns to 1000 ns was investigated. The simulation results are shown in Figure 10, where black points represent the damage threshold obtained from the simulation calculation and the red line represents the fitted curve. It can be seen that peak power density *P*_0_ presents an obvious inverse proportional relationship with pulse width. Equation (19) provides a functional relationship between *P*_0_ and pulse width *τ*. When *τ* is far less than 1000 ns, the energy density is close to a fixed value of about 0.51 Jcm^−2^. This relationship is valuable for predicting the damage threshold of HgCdTe FPA detectors irradiated using mid-infrared laser with other pulse widths.

(19) $$P_{0}=\frac{0.51}{\tau }$$

## 4. Discussion

*E_c_* was observed to be marginally lower than *E*_0_. Further analysis revealed several factors that contribute to this discrepancy.

- The actual detector structure is much more intricate than the theoretical model, necessitating simplification of the model. The calculation accuracy, ability, and model matching degree must be balanced to ensure minimal deviation in the results.
- The loading method of light source differs from the actual situation. In practice, the laser irradiates the detector surface through a lens, which introduces additional factors such as lens transmissivity and detector reflection. These factors are not considered in the theoretical model, leading to a higher measured energy before the lens than the energy absorbed by the detector. Consequently, the experimental damage threshold *E*_0_ is expected to be higher than the theoretical damage threshold *E_c_*.
- There may be discrepancies in the physical parameters of materials that cannot be accounted for in the theoretical model.

Despite these challenges, the damage threshold calculated via simulation is generally consistent with *E*_0_, and the error between the two is acceptable. Thus, the model is deemed reliable and can serve as a theoretical basis for studying damage effects of mid-infrared HgCdTe FPAs detector.

In the future, we hope to use scanning electron microscopy and electronic detection equipment to study the inherent mechanisms of damage to morphology, leakage current, and circuit of the damaged detector chips.

## 5. Conclusions

In summary, experimental and simulation studies on a HgCdTe FPA detector damaged using a mid-infrared pulsed laser were conducted. The experimental data of point damage, line damage, and full-frame damage of the detector were obtained. The results showed that experimental damage threshold *E*_0_ of the HgCdTe FPA detector is within the range of 0.59–1.17 Jcm^−2^. A damage model for laser-irradiated HgCdTe FPA detectors was established. By setting the same wavelength, pulse width, and detector pixel size as in the experiment, the heat transfer mechanism, stress, and displacement deformation inside the detector were studied. The simulation results indicated that before HgCdTe reaches the melting point, the maximum internal stress occurs at the interface between CdZnTe and HgCdTe. The magnitude of the displacement deformation can be ignored. By comparing temperature changes in HgCdTe and indium, it was found that the HgCdTe layer reached its melting point earlier. Therefore, based on the melting damage of the HgCdTe layer, the damage threshold at a pulse width of 30 ns was obtained, which is *E_c_* = 0.55 Jcm^−2^. We studied the damage threshold under other pulse widths and found that the damage threshold characterized by peak power density exhibits an inverse proportional change with pulse width when the pulse width is less than 1 μs. At this point, the energy density approaches a fixed value of 0.51 Jcm^−2^. Finally, by analyzing the model, the error between the experimental and simulation results is acceptable. The research results in this paper can be used to guide detector protection, predict experimental damage thresholds under other pulse widths, and optimize chip processing.

## Figures and Tables

**Table 1:** Physical parameters of each layer of material.

Physical Parameter	CdZnTe	HgCdTe	Indium	Si
Density ρ (gcm^−3^)	5.68	7.64	7.3	2.33
Specific heat c (Jg^−1^K^−1^)	0.159	c(T)	0.233	0.7
Thermal conductivity *k* (Wcm^−1^K^−1^)	0.01	k(T)=K(T)ρc(T)	0.82	1.3
Coefficient of thermal expansion *β* (K^−1^)	5 × 10^−6^	5 × 10^−6^	3.3 × 10^−5^	1.15 × 10^−6^
melting point (K)	1460	993	426	1410
Elastic module *E* (Pa)	3.98 × 10^11^	5 × 10^10^	1.06 × 10^10^	1.3 × 10^11^
Poisson’s ratio	0.459	0.31	0.45	0.24

## Data Availability

The data presented in this study are available on request from the corresponding author.
